# Cardiorespiratory Fitness Is Associated with Hard and Light Intensity Physical Activity but Not Time Spent Sedentary in 10–14 Year Old Schoolchildren: The HAPPY Study

**DOI:** 10.1371/journal.pone.0061073

**Published:** 2013-04-05

**Authors:** Sarah J. Denton, Michael I. Trenell, Thomas Plötz, Louise A. Savory, Daniel P. Bailey, Catherine J. Kerr

**Affiliations:** 1 Institute for Sport and Physical Activity Research, University of Bedfordshire, Bedford, United Kingdom; 2 Institute of Cellular Medicine, Newcastle University, Newcastle upon Tyne, United Kingdom; 3 School of Computing, Newcastle University, Newcastle upon Tyne, United Kingdom; Pennington Biomedical Research Center, United States of America

## Abstract

**Background:**

Sedentary behaviour is a major risk factor for developing chronic diseases and is associated with low cardiorespiratory fitness in adults. It remains unclear how sedentary behaviour and different physical activity subcomponents are related to cardiorespiratory fitness in children. The purpose of this study was to assess how sedentary behaviour and different physical activity subcomponents are associated with 10–14 year-old schoolchildren's cardiorespiratory fitness.

**Methods:**

135 schoolchildren (81 girls, 12±1 year) completed 7-day minute-by-minute habitual physical activity monitoring using triaxial accelerometers and undertook a maximal cardiorespiratory fitness test.

**Results:**

After controlling for sex, age, ethnicity, socioeconomic status and total wear time, light physical activity (1.5–2.9 METs) was negatively associated (*β* = −.24, *p*<.01) and hard physical activity (≥9 METs) positively associated (*β* = .45, *p*<.001) with cardiorespiratory fitness. Vigorous and hard physical activity were associated with cardiorespiratory fitness for boys (*F* = 5.64, *p*<.01) whereas light, moderate and hard physical activity were associated with physical fitness for girls (*F* = 10.23, *p*<.001). No association was found between sedentary time and cardiorespiratory fitness (*r* = −.13, *p*>.05). Sedentary to active transitions revealed little variability between cardiorespiratory fitness tertiles.

**Conclusions:**

Hard physical activity (≥9 METs) holds greater potential for cardiorespiratory fitness compared to physical activity of lower intensities. There was no relationship between sedentary behaviour and cardiorespiratory fitness. These findings suggest that, for children, advice should focus on higher intensity physical activity and not sedentary behaviour as a means to maintain or improve cardiorespiratory fitness. Future research should explore longitudinal relationships between hard physical activity, cardiorespiratory fitness and health parameters.

## Introduction

Low cardiorespiratory fitness is an independent risk factor for cardiovascular disease, type 2 diabetes mellitus, and mortality in adults [1] and is unfavourably associated with cardiometabolic risk factors in children and adolescents [2], [3]. Inverse relationships exist between cardiorespiratory fitness and clustered cardiovascular disease risk scores in European youths (n = 2845) [4] and U.S adolescents (n = 1247) [5]. Physical activity and cardiorespiratory fitness are closely related in adults [1]. Pursuing a physically active lifestyle is positively associated with many physiological and psychological health benefits [6] but engagement in this behaviour is low in children, with a large percentage of both boys and girls (67% and 79%, respectively) aged 4–15 years not achieving the recommended levels of physical activity in England [7].

Vigorous physical activity (or higher) is more strongly associated with both cardiorespiratory fitness [8], [9], [10] and body fatness in children compared to lower intensities, such as moderate physical activity [11], [12], [13]. Time spent sedentary presents an additional potential risk factor to the development of disease [14]. Indeed, recently updated guidelines [15] highlight that children should minimise the amount of time spent sedentary due to the associated risk with a number of chronic diseases [14]. Sedentary bout accumulation has been previously examined in adults by Power Law Analyses with the most inactive accumulating sedentary time over longer bouts [16].

Few studies have offered a detailed analysis of the associations of sedentary behaviours and physical activity subcomponents with cardiorespiratory fitness and rarely have investigators reported physical activity intensities above vigorous [10]. The potential impact of higher intensities, such as ‘hard intensity’ physical activity (equivalent to ≥9 METs [17]) on cardiorespiratory fitness, requires further investigation. Furthermore, no studies to the author's knowledge have considered Power Law Analyses when understanding sedentary bout accumulation as well as transition from sedentary behaviour to an active subcomponent in children. The purpose of this study was therefore to provide a more detailed insight into the importance and relative contributions of sedentary behaviour and the different physical activity subcomponents to cardiorespiratory fitness in 10–14 year-old schoolchildren.

## Materials and Methods

### Ethics Statement

The Health And Physical activity Promotion in Youth study (the HAPPY study) received full ethical approval from the University of Bedfordshire ethics review board. Written informed consent was obtained from participants' parents and verbal assent from the participants before any testing procedures.

### Sample

A subset of 135 HAPPY study participant's baseline data (81 girls, 12±1 year) were included in this study as they met the accelerometry inclusion criteria and completed the maximal cardiorespiratory fitness test to exhaustion. Primary aims of this school-based intervention study were to increase overall time spent in moderate-vigorous physical activity during school and leisure time, and improve cardiorespiratory fitness, physical health and psychosocial well-being in 10–14 year-old children. A total of 249 participants were recruited on a voluntary basis from 11 middle and upper schools in Bedfordshire, United Kingdom. Participants were excluded if they had any contraindications to taking part in physical exercise.

### Measurements

Age was recorded as a decimal value for each participant on the day of data collection. Ethnicity was recorded as white or non-white. Socioeconomic status was calculated from the 2007 Indices of Multiple Deprivation from each participant's postcode [18] and were categorised into tertiles with the lowest tertile indicating the most deprived. Standing height was measured to the nearest 0.5 cm using the portable Leicester Height Measure (Seca, Birmingham). Body weight was measured to the nearest 0.1 kg using the Tanita BC-418® (Tanita Corp., Tokyo). Body mass index (BMI) was calculated as body mass (kg)÷standing height^2^ (m^2^). UK 1990 reference values were used to calculate z-scores for BMI [19], [20].

RT3® triaxial accelerometers (Stayhealthy, Inc., Monrovia, CA) were employed to measure 7 day minute-by-minute habitual physical activity monitoring. This monitor has previously demonstrated good reliability (coefficient of variation = 0.29% to 1.81%) at acceleration frequencies of 2.5 Hz and 4.6 Hz [21] and strong validity against oxygen consumption in children (*r* = .87, *p*<.01) [17]. The Rowlands et al (2004) [17] cut-off thresholds were used to determine time spent in each subcomponent, which included sedentary behaviour (<288 counts per minute; <1.5 METs), light physical activity (288–969 counts per minute; ≥1.5 METs), moderate physical activity (970–2332 counts per minute; ≥3 METs), vigorous physical activity (2333–3200 counts per minute; ≥6 METs) and hard physical activity (≥3201 counts per minute; ≥9 METs). The inclusion criteria were a minimum wear time of three days [22], [23] and a minimum daily wear time of nine hours for weekdays [22] and eight hours for weekend days [24]. Data were checked for non-wear time and sustained 10 minute periods of zero counts were removed during the recoding process [25].

To determine cardiorespiratory fitness, participants completed an age- and sex-specific all-out progressive cycle ergometer test to exhaustion using a previously validated protocol [26]. Workloads increased every 3 minutes until the participant was no longer able to continue. A maximal effort was deemed as a final heart rate ≥185 beats per minute and subjective observation from the researcher that the child could not continue [26]. Power output (watts) was calculated as being equal to W_1_+(W_2_^.^ t/180), where W_1_ is work rate at fully completed stage, W_2_ is the work rate increment at final incomplete stage, and t is time in seconds at final incomplete stage. VO_2max_ was calculated using previously described formulae [26] and expressed relative to fat free mass (mL.kgFFM.min^−1^) to account for between-individual differences in body size [27].

### Statistical analysis

Prior to analysis, procedures for checking violations of test assumptions were conducted. Both vigorous and hard physical activity subcomponents were non-normally distributed and were subsequently square-root transformed for the main analyses [28]. Descriptive statistics were presented as mean±SD. Independent t-tests assessed sex differences for all measured variables. Bivariate correlation analyses using Pearson assessed correlations between the subcomponents (sedentary behaviour and physical activity subcomponents) and cardiorespiratory fitness. A multiple regression analysis using the forward method established which subcomponents (sedentary behaviour and physical activity) were significantly associated with cardiorespiratory fitness after controlling for sex, age, ethnicity, socioeconomic status and total wear time. Follow-up multiple regression analyses using the forward method were performed split by sex. Patterns of sedentary time accumulation were determined between tertiles of cardiorespiratory fitness (low = ≤47.5; middle = <56; high = >56 mL.kgFFM.min^−1^, respectively) using Power Law Analyses [16]. Sedentary to active transitions were calculated from raw minute-by-minute accelerometry data to determine the length of sedentary bout and the amount of crossovers from sedentary behaviour to active subcomponents (the sedentary to active transitions). All data were analysed using SPSS, version 19 (SPSS Inc., Chicago, IL, USA). The level of significance was set at *p*<.05.

## Results

Descriptive statistics are detailed in Table 1 for all participants and by sex. Girls spent more time in light physical activity than boys, whereas boys accumulated more time in vigorous and hard physical activity and had higher cardiorespiratory fitness scores than girls (*p*<.01). No significant sex differences were found for age, standing height, body weight, zBMI or time spent sedentary or in moderate physical activity. No significant differences were found between the percentage of boys and girls achieving the levels of physical activity stipulated in the government guidelines [15], [29].

**Table 1:** Physical characteristics and time spent sedentary and in the different physical activity subcomponents.

Variables	All (n = 135)	Boys (n = 54)	Girls (n = 81)
Age (years)	12±1	12±1	12±1
Ethnicity (% non-white)	24	24	24
Standing height (cm)	150±11	151±12	150±9
Body weight (kg)	41±12	40±12	41±12
zBMI	−0.3±1.4	−0.4±1.3	−0.3±1.4
Cardiorespiratory fitness (mL.kgFFM.min^−1^)	52±10	56±8*	50±10
Sedentary behaviour (minutes/day, <1.5 METs)	452±75	440±70	461±78
Light physical activity (minutes/day, 1.5–2.9 METs)	178±43	165±34*	186±47
Moderate physical activity (minutes/day, 3–5.9 METs)	84±30	87±27	81±31
Vigorous physical activity (minutes/day, 6–8.9 METs)+	14±10	17±9*	12±10
Hard physical activity (minutes/day, ≥9 METs)+	8±9	11±10*	6±7
Total wear time (minutes/day)	736±67	721±63	747±67
Meeting physical activity guidelines (%)	90	94	88

Notes:

* Significant difference between sexes (*p*<.01).

+ Square-root transformed in main analyses, untransformed data displayed.

Simple correlations between the subcomponents (sedentary and physical activity) and cardiorespiratory fitness are detailed in Table 2 and revealed that light physical activity was inversely correlated whereas moderate, vigorous and hard physical activity were all positively correlated with cardiorespiratory fitness. For boys, light physical activity was negatively correlated and hard physical activity positively correlated with cardiorespiratory fitness. For girls, moderate, vigorous and hard physical activity were all positively correlated with the outcome variable. Table 3 provides details of the correlations between each subcomponent.

**Table 2:** Correlations between cardiorespiratory fitness and both sedentary behaviour and the physical activity subcomponents.

	Cardiorespiratory fitness		
	All	Boys	Girls
Sedentary behaviour (<1.5 METs)	−.13	.00	−.14
Light physical activity (1.5–2.9 METs)	−.23**	−.28*	−.13
Moderate physical activity (3–5.9 METs)	.17*	−.15	.29**
Vigorous physical activity (6–8.9 METs)	.33***	.05	.37***
Hard physical activity (≥9 METs)	.44***	.30*	.44***
Total wear time (minutes/day)	−.11	−.15	−.02

Notes:

* *p*<.05;

** *p*<.01;

*** *p*<.001.

**Table 3:** Pearson correlations between each of the subcomponents.

		1	2	3	4	5
All	1. Sedentary (<1.5 METs)		−.42***	−.53***	−.43***	−.37***
Boys			−.43**	−.36**	−.38**	−.38**
Girls			−.49***	−.61***	−.43***	−.32**
All	2. Light physical activity (1.5–2.9 METs)	−.42***		.48***	.14	.02
Boys		−.43**		.55***	.11	−.09
Girls		−.49***		.51***	.28**	.22*
All	3. Moderate physical activity (3–5.9 METs)	−.53***	.48***		.63***	.39***
Boys		−.36**	.55***		.41**	.15
Girls		−.61***	.51***		.74***	.54***
All	4. +Vigorous physical activity (6–8.9 METs)	−.43***	.14	.63***		.81***
Boys		−.38**	.11	.41**		.79***
Girls		−.43***	.28**	.74***		.80***
All	5. +Hard physical activity (≥9 METs)	−.37***	.02	.39***	.81***	
Boys		−.38**	−.09	.15	.79***	
Girls		−.32**	.22*	.54***	.80***	

Notes:

* *p*<.05;

** *p*<.01;

*** *p*<.001.

+ Square-root transformed in main analyses.

The multiple regression analysis (all participants) revealed that both light and hard physical activity were significantly associated with cardiorespiratory fitness (*F* = 22.18, *p*<.001), accounting for 25% of the variance. As shown in Table 4, light physical activity was negatively associated and hard physical activity positively associated with cardiorespiratory fitness. All other variables did not explain a significant variation in cardiorespiratory fitness scores.

**Table 4:** Multiple regression analyses using the forward method examining the association of cardiorespiratory fitness with sedentary behaviour and physical activity subcomponents for all participants and sex-specific.

	Unstandardised Coefficients		Standardised
	*B*	*SE B*	*B*
All			
Light physical activity (1.5–2.9 METs)	−.05	.02	−.24**
Hard physical activity (≥9 METs)	3.06	.52	.45***
Boys			
Vigorous physical activity (6–8.9 METs)	−3.70	1.55	−.50*
Hard physical activity (≥9 METs)	4.02	1.21	.69**
Girls			
Light physical activity (1.5–2.9 METs)	−.07	.02	−.35**
Moderate physical activity (3–5.9 METs)	.08	.04	.27*
Hard physical activity (≥9 METs)	2.92	.90	.37**

Notes:

* *p*<.05;

** *p*<.01;

*** *p*<.001.

Table 4 details the separate sex-specific regression analyses, which found that for boys, vigorous and hard intensity physical activity were significant coefficients in the model (*β* = 5.64, *p*<.01) accounting for 18% of the variance in physical fitness. For girls, light, moderate and hard physical activity were significantly associated with cardiorespiratory fitness scores (*F* = 10.23, *p* = <.001), accounting for 29% variance with the coefficients demonstrating similar directions as the correlation analysis.

**Figure 1:**
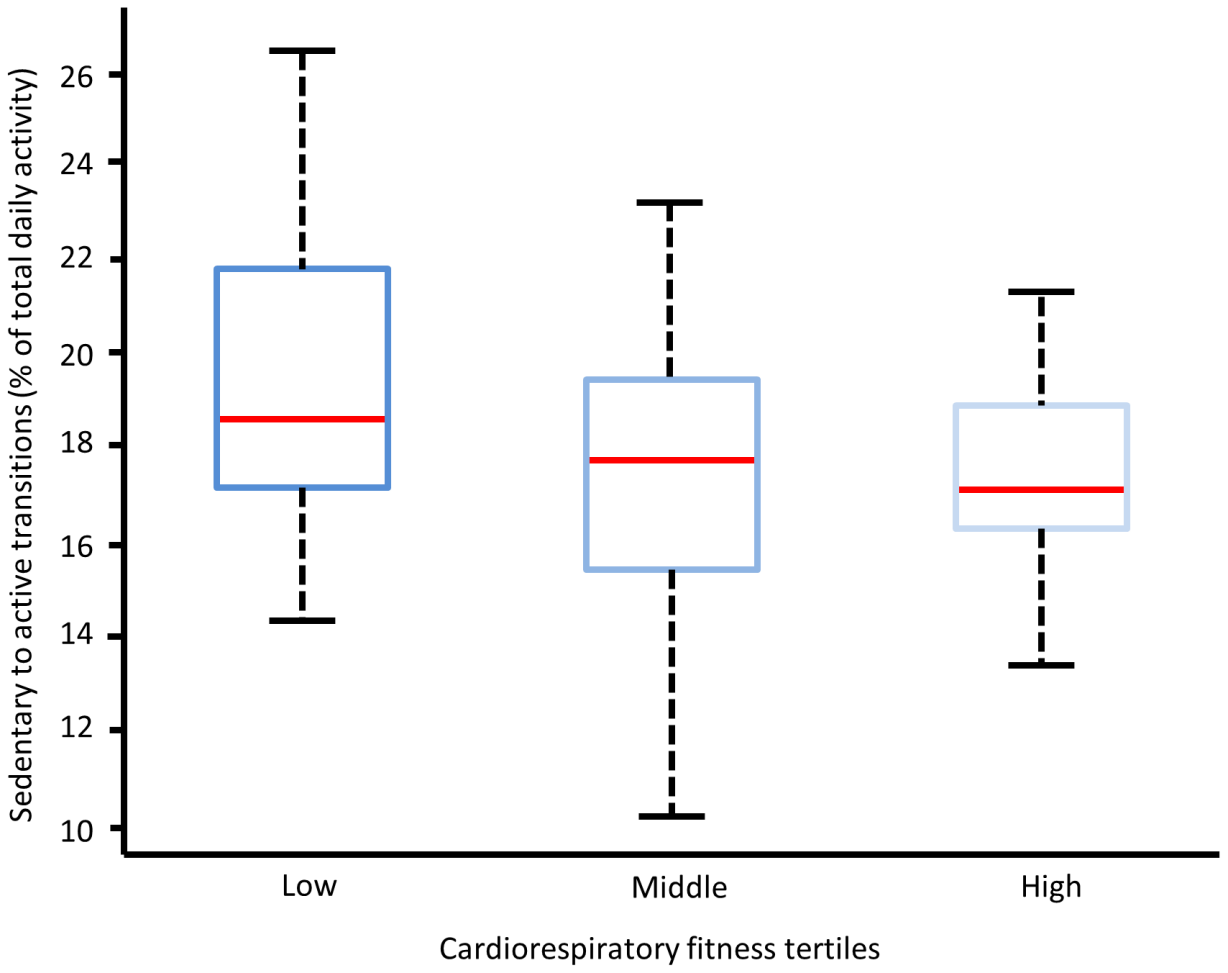
Sedentary to active transitions for participants in low, middle and high cardiorespiratory fitness tertiles (low = ≤47.5; middle = <56; high = >56 mL.kgFFM.min^−1^, respectively).

**Figure 2:**
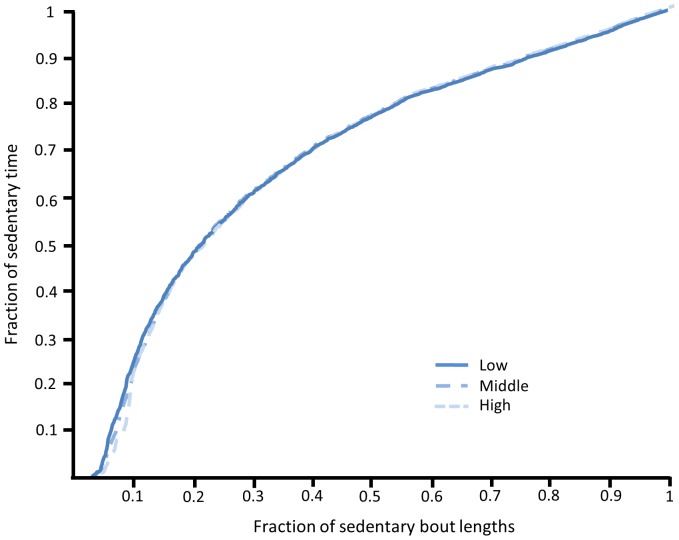
Sedentary bout lengths plotted against the fraction of total sedentary time for participants in the low, middle and high cardiorespiratory fitness tertiles (low = ≤47.5; middle = <56; high = >56 mL.kgFFM.min^−1^, respectively).

Patterns of sedentary time between tertiles of cardiorespiratory fitness are displayed in Figures 1 and 2. Figure 1 details the transitions from a sedentary behaviour to an active subcomponent with the box plots displaying small variability between the three groups. Similarly, the average length of each sedentary bout by the total sedentary time from the raw accelerometry counts is displayed in Figure 2 with the traces indicating little difference between cardiorespiratory fitness groups.

## Discussion

This is the first study to define the relationship between cardiorespiratory fitness and both sedentary behaviour and a wide span of activity intensities that includes hard intensity physical activity in children. The data demonstrate that hard physical activity (≥9 METs) explained a significant proportion of the variance in cardiorespiratory fitness, while time spent sedentary and in moderate and vigorous physical activity did not. Time spent in light physical activity was significantly inversely associated with cardiorespiratory fitness. This study found both light and hard intensity physical activity were associated with current cardiorespiratory fitness in 10–14 year-old schoolchildren.

Positive associations found between time spent in higher intensity physical activity and cardiorespiratory fitness is in agreement with previous research in similar aged youths to this current study [8], [10], [12], [30]. Interestingly, when the analysis was separated by sex, vigorous and hard physical activity were the significant coefficients for boys whereas for girls, light, moderate and hard physical activity levels were significantly associated with physical fitness.

Cardiorespiratory fitness is associated with cardiovascular disease risk in children and adolescents [4]; therefore, opportunities should be provided to promote engagement in higher intensity physical activity such as ≥9 METs (hard physical activity) as emphasised in this study. This level of activity is estimated during short, sporadic bursts of movement involving a hard effort, for example, basketball dribbling and shooting or skipping and jumping, which are encountered during everyday physical activities [31]. However, it has previously been reported that not all children will be able to achieve and maintain this level of physical activity [32]. The school setting provides an ideal environment for engagement in physical activity [33] but the structure of the school day may restrict the level of physical activity needed to improve cardiorespiratory fitness [34] due to the prolonged periods of lesson time spent sedentary [35].

The general decline in physical activity in childhood is associated with an increased incidence of obesity [7]. Obesity is a recognised cardiovascular disease risk factor and inverse relationships exist between time spent in higher intensity physical activity and body fat [29], [36]. Previous studies have shown that time spent in both vigorous and hard physical activity are inversely associated with body fat (*r* = −.44, *p*<.004 and *r* = −.39, *p*<.014, respectively) but moderate physical activity is not [11]. Higher cardiorespiratory fitness scores are associated with lower relative risk of being overweight/obese (odds ratio = .97, *P* = .04) versus lower scores [10], which makes the positive associations found in this study for both boys and girls between hard intensity physical activity and cardiorespiratory fitness particularly important.

In contrast to the observed associations between higher intensities of physical activity and cardiorespiratory fitness, the negative associations found between light physical activity and cardiorespiratory fitness indicate that spending time in this intensity does not benefit cardiorespiratory fitness in this age group. However, when the regression analysis was separated by sex, these negative associations were only significant for girls. It is likely that light physical activity is replacing time spent in higher intensity physical activities and this is having a negative effect on cardiorespiratory fitness scores as demonstrated by lower scores for girls versus boys. This observation suggests that engagement in light physical activity is detrimental to health, but only as it represents a lack of more intense physical activity [37]. Previous research has found light physical activity (1.5–2.9 METs) to be non-significant when associated with cardiorespiratory fitness [10]. Interestingly, this variable was not included in a previous study examining associations between sedentary behaviour, physical activity subcomponents (moderate and vigorous physical activity), cardiorespiratory fitness and blood pressure [38]. Given the significant sex differences in time spent in light physical activity in this current study (165±34 vs. 186±47 minutes/day, *p*<.01, boys vs. girls, respectively), these findings highlight that time spent in this intensity has a negative association with cardiorespiratory fitness for girls but not boys.

These current findings highlight that schoolchildren should be encouraged to participate in higher intensity physical activity (≥9 METs) given the overall positive associations found between this intensity and cardiorespiratory fitness. For boys, this intensity was the only variable to significantly correlate with fitness whereas for girls, moderate physical activity or above (i.e. vigorous and hard physical activity) was positively correlated with cardiorespiratory fitness. The health benefits of schoolchildren achieving the minimum levels of physical activity stipulated in the government guidelines has been previously reiterated [15], [29], however, these findings reinforce the need to promote engagement in higher intensity physical activity for health. Because of the cross-sectional nature of this study, reverse causation cannot be dismissed. However, prospective studies have demonstrated that performing higher intensity physical activity over a prolonged period has led to increases in cardiorespiratory fitness [32] and this should be explored further in future studies.

Previous reports suggest that children should be encouraged to minimise the amount of time spent sedentary due to the increased risk of chronic diseases [14], [15]. However, the present study demonstrates that in this sample of children neither the duration of sedentary behaviour nor the breaks in sedentary behaviour (sedentary to active transitions) were associated with cardiorespiratory fitness. Interestingly, the data suggests that 10–14 year-old schoolchildren have similar sedentary time regardless of cardiorespiratory fitness scores, possibly due to the structured and standardised nature of school days between children. No associations were found between sedentary behaviour and BMI (all participants, *r* = .05, *p*>.05). Therefore, the similarities in sedentary behaviour between children highlights the importance of promoting higher intensity physical activity to maintain cardiorespiratory fitness and to reduce the incidence of clustered cardiovascular disease risk [4], [5]. However, the accelerometry inclusion criteria (a minimum of nine hours monitoring on weekdays) [22] may not have captured all sedentary behaviour and limited the differences found between groups.

This study was not without limitations, which include the cross-sectional nature of this study, which mean causal inferences cannot be made. The relatively small sample makes it difficult to generalise findings. However, the sample may still be representative of other areas in England according to the Child Well-Being Index scores of childhood deprivation [39]. The sample was self-selected and participants were required to achieve a minimum accelerometry wear time criteria to be included in the analysis, which may have biased the sample towards participants who engaged in physical activity and excluded those who were disengaged in this behaviour. Minute-by-minute accelerometry sampling intervals were employed, which may have led to underreporting of more sporadic, short bursts of higher intensity activities due to a combination of both high and low intensity activity being captured within the same epoch [40], [41]. However, the equipment (RT3® triaxial accelerometer) employed meant only this sampling frequency could be used to measure 7 consecutive days of habitual physical activity.

In conclusion, these data demonstrate that when considering the role of moderate, vigorous and hard physical activity in promoting cardiorespiratory fitness, hard physical activity (≥9 METs) is of particular importance in 10–14 year-old schoolchildren. The negative association found between light physical activity and cardiorespiratory fitness reinforces the importance of children engaging in higher intensity physical activity to benefit health and well-being. The total amount of sedentary behaviour or breaks in sedentary behaviour were not associated with cardiorespiratory fitness or BMI. Future intervention studies should consider a particular emphasis on hard intensity physical activities when promoting physical activity opportunities, where appropriate.
