# Correction: Cardiorespiratory Fitness Is Associated with Hard and Light Intensity Physical Activity but Not Time Spent Sedentary in 10–14 Year Old Schoolchildren: The HAPPY Study

**DOI:** 10.1371/annotation/09abd773-39df-46a9-b28e-3031be04fcb9

**Published:** 2013-10-08

**Authors:** Sarah J. Denton, Michael I. Trenell, Thomas Plötz, Louise A. Savory, Daniel P. Bailey, Catherine J. Kerr

There were multiple errors in the Author Contributions statement. The Author Contributions statement should read: "Conceived and designed the experiments: SJD LAS DPB CJK. Performed the experiments: SJD LAS DPB CJK.

Analyzed the data: SJD TP LAS DPB CJK. Wrote the paper: SJD MIT TP LAS DPB CJK." 

